# Mesenchymal stem cells as therapeutic target of biophysical stimulation for the treatment of musculoskeletal disorders

**DOI:** 10.1186/s13018-016-0496-5

**Published:** 2016-12-16

**Authors:** Marco Viganò, Valerio Sansone, Maria Cristina d’Agostino, Pietro Romeo, Carlotta Perucca Orfei, Laura de Girolamo

**Affiliations:** 1 IRCCS Galeazzi Orthopaedic Institute, Via R. Galeazzi 4, 20161 Milan, Italy; 2 Department of Biotechnology and Biosciences, University of Milano-Bicocca, Milan, Italy; 3 Department of Biomedical Science for Health, University of Milan, Milan, Italy; 4 ESWT Center, Rehabilitation Department, Humanitas Research Hospital, Rozzano, Milan, Italy; 5 Department of Drug Sciences, University of Pavia, Pavia, Italy

**Keywords:** Mesenchymal stem cells, Pericytes, Electromagnetic fields, PEMF, Shock waves, ESWT

## Abstract

**Background:**

Musculoskeletal disorders are regarded as a major cause of worldwide morbidity and disability, and they result in huge costs for national health care systems. Traditional therapies frequently turned out to be poorly effective in treating bone, cartilage, and tendon disorders or joint degeneration. As a consequence, the development of novel biological therapies that can treat more effectively these conditions should be the highest priority in regenerative medicine.

**Main body of the abstract:**

Mesenchymal stem cells (MSCs) represent one of the most promising tools in musculoskeletal tissue regenerative medicine, thanks to their proliferation and differentiation potential and their immunomodulatory and trophic ability. Indeed, MSC-based approaches have been proposed for the treatment of almost all orthopedic conditions, starting from different cell sources, alone or in combination with scaffolds and growth factors, and in one-step or two-step procedures. While all these approaches would require cell harvesting and transplantation, the possibility to stimulate the endogenous MSCs to enhance their tissue homeostasis activity represents a less-invasive and cost-effective therapeutic strategy. Nowadays, the role of tissue-specific resident stem cells as possible therapeutic target in degenerative pathologies is underinvestigated. Biophysical stimulations, and in particular extracorporeal shock waves treatment and pulsed electromagnetic fields, are able to induce proliferation and support differentiation of MSCs from different origins and affect their paracrine production of growth factors and cytokines.

**Short conclusions:**

The present review reports the attempts to exploit the resident stem cell potential in musculoskeletal pathologies, highlighting the role of MSCs as therapeutic target of currently applied biophysical treatments.

## Background

The physiological role of mesenchymal stem/stromal cells (MSCs) is the maintenance of tissue homeostasis, mainly by proliferation and differentiation toward mature tissue specific cell types, and also through the release of growth factors and immunomodulatory agents [1].

Indeed, the pathological tissue degeneration, which occurs in most of the orthopedic disorders, follows an unbalance in tissue homeostasis, due to inflammation, overuse, or trauma. Therefore, the MSCs failure in maintaining this homeostasis may represent the first step in the development of many orthopedic pathologies. On these premises it is possible to hypothesize that a direct therapeutic action aimed to enhance MSCs activity could revert the progression of degenerative disorders.

In the last decades, MSCs have emerged as a possible powerful tool in the treatment of various diseases related to tissue degeneration, inflammation, and trauma.

In 2006, when the exact location and function within the native tissues was not fully understood yet, the International Society for Cellular Therapy released a definition of MSCs that included their propensity to adhere to polystyrene (plastic) in in vitro culture; the expression of CD105, CD73, and CD90 surface antigens; and the ability to differentiate, under appropriate stimuli, at least toward osteoblasts, adipocytes, and chondroblasts cell lines [2]. In addition to bone marrow, which is the most traditional source, MSCs have been isolated from almost all body compartments [3], including tendons [4], periosteum [5], trabecular bone [6], adipose tissue [7], synovial membrane [8], and muscle [9]. This multilocalization of MSCs relies on their origin; indeed, it is now well established that MSCs, or their precursors, lie in perivascular locations in all the vascularized tissues [10]. Accurate studies have demonstrated that microvascular pericytes [10] and adventitial cells [11], both freshly isolated or expanded in culture, are indistinguishable from conventional MSCs; hence, the terms perivascular stem cells or pericytes (PSCs) have been applied to these cells as well.

In addition to their ability to differentiate and to participate directly to the regeneration process, MSCs present further therapeutic functions in response to injury. Indeed, in response to particular conditions, MSCs are able to release a plethora of cytokines and growth factors with immunomodulatory and trophic effect on the pathological tissues [1]. Immunomodulatory activity is mediated by direct cell-cell contact and through secreted bioactive molecules involving dendritic cells, B and T cells [12], whereas trophic effects of MSCs are based on the secretion of molecules that inhibit apoptosis and fibrosis, and the stimulation of angiogenesis through the secretion of vascular endothelial growth factor (VEGF) [13]. For all these reasons they have been recently called "drugstores" [1].

All these features support the application of MSCs to a wide range of pathologies characterized by inflammation and degeneration, either alone [14] or in combination with scaffolds [15], the latter especially when used in tissue engineering applications to obtain artificial biologic substitutes.

## Mesenchymal stem cells: a multimodal tool to treat musculoskeletal diseases

The therapeutic potential of MSCs have been largely investigated in in vitro and preclinical settings, as well as in many studies at clinical level, for their ability to improve healing from orthopedic conditions such as chondral lesion [16], osteoarthritis [17], tendinopathy [18], intervertebral disk disease [19], and bone non-union/delayed unions [20]. MSCs have been used either expanded [21] or as concentrated progenitor pools [22], both with satisfactory results.

Even if further studies are needed to confirm their utility and to standardize applications, the results of the use of MSCs in orthopedic conditions are very promising and many studies are nowadays ongoing [17].

On the contrary, the possibility to stimulate resident MSCs in order to improve physiological tissue healing is relatively innovative, and very few studies are reported in literature on this topic so far. This idea is supported by different studies conducted in pathological conditions demonstrating that MSCs, exposed to damage associated molecular pattern (DAMP) or pathogen-associated molecular pattern (PAMP), could react by reverting the production of pro-inflammatory to anti-inflammatory molecules [23, 24] and by inducing the switch from pro-inflammatory M1 macrophages maturation to the anti-inflammatory M2 phenotype [25]. These observations open the field to the hypothesis that an enhancement of MSCs potential would benefit the progress of many orthopedic conditions. Their plasticity and ability to respond to external stimuli, both physical and chemical, make MSCs a very good target for different treatments intended to improve tissue regeneration.

The purpose of this work is to review the attempts to exploit the resident stem cell potential in musculoskeletal-related pathologies, in order to highlight their function as therapeutic target. In particular, we will focus on biophysical stimulation, such as shock waves and pulsed electromagnetic fields, since they are already applied in clinical practice, and the recent advancement in the knowledge about their mechanism of action supports the hypothesis that they would be effective in the stimulation of endogenous MSCs action (Table 1).

**Table 1:** In vitro effects of PEMF and SW on MSCs from different origins

MSCs origin	PEMF	ESWT
Bone marrow	Increased proliferation [40–43]	Increased proliferation [63]
	Enhanced osteogenic [41, 42, 44, 45] and chondrogenic [51] differentiation	Increased migration [63]
	Reduced production of inflammatory mediators [27]	Enhanced osteogenic differentiation [61–63]
Adipose tissue	Increased proliferation [52]	Enhanced osteogenic [71, 72] and adipogenic [72] differentiation
	Enhanced chondrogenic [53] differentiation	Increased migratory ability [70]
Tendon tissue	Increased expression of tissue specific markers [56]	Increased expression of tissue specific markers [68, 69]
	Increased production of trophic and anti-inflammatory mediators [56, 57]	Increased production of trophic and anti-inflammatory mediators [69]
Umbilical cord	Enhanced chondrogenic [54] differentiation	—
Endothelial tissue	—	Increased proliferation [64]
		Enhanced migration and homing to lesion sites [64, 65]

## Pulsed electromagnetic field

**Fig. 1:**
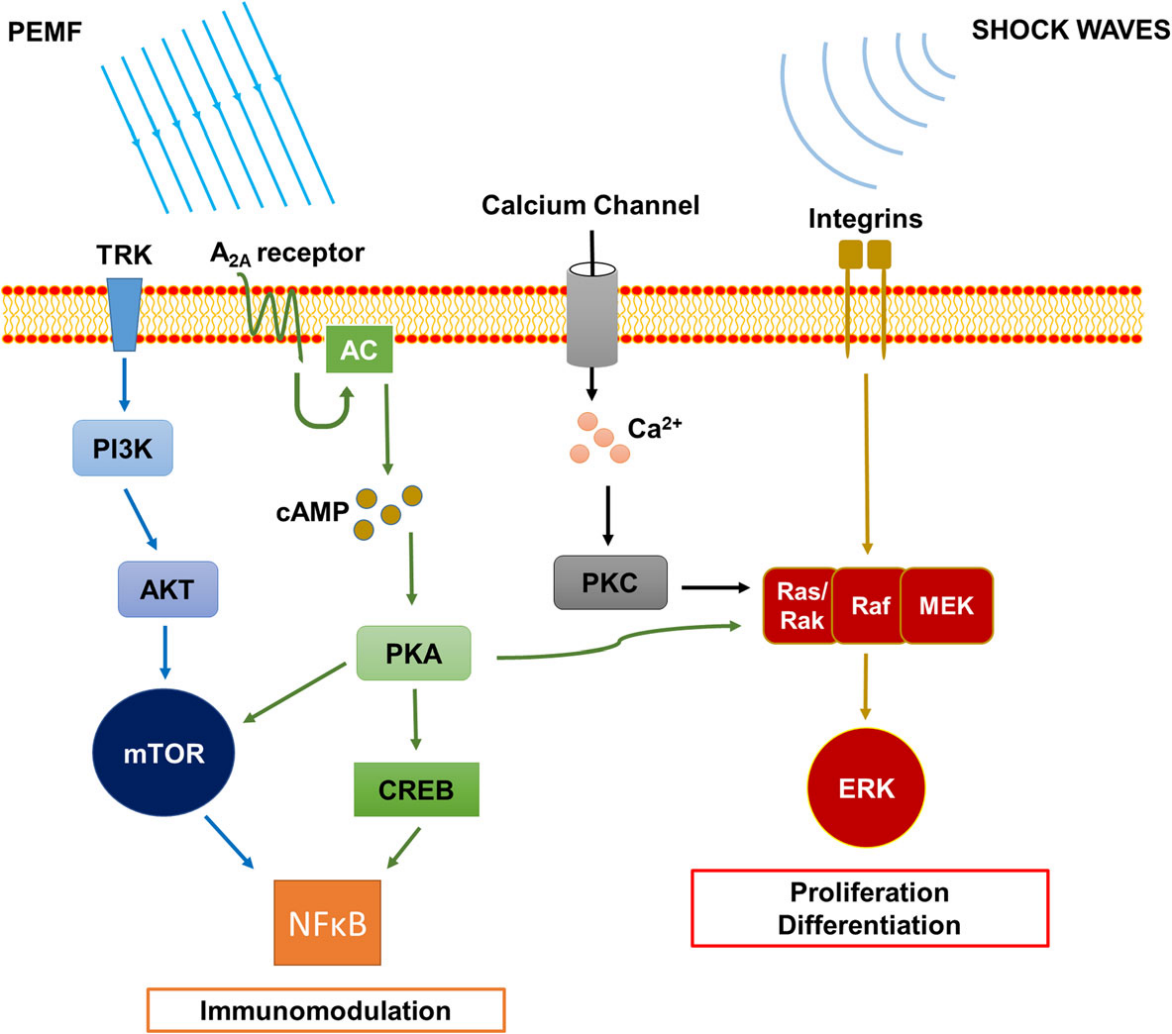
Possible molecular pathways involved in the biological response to PEMFs and ESWT stimulations. Biophysical stimulations could act through ERK and mTOR pathways to enhance cell proliferation and differentiation and to modulate the inflammatory response. *TRK* tyrosine kinase receptor, *PI3K* phosphatidylinositide 3-kinases, *PKB* protein kinase B (also known as AKT), *mTOR* mechanistic target of rapamycin, *NF-*_*K*_ *B* nuclear factor kappa-light-chain-enhancer of activated B cells, *AC* adenylyl cyclase, *cAMP* cyclic adenosine monophosphate, *PKA* protein kinase A, *CREB* cAMP response element-binding protein, *PKC* protein kinase C, *Rac*/*Ras* small GTPase of the Ras superfamily, *Raf* serine/threonine-specific protein kinases. *MEK* mitogen-activated protein kinase kinase, and *ERK* extracellular signal-regulated kinases

Pulsed electromagnetic fields (PEMFs) are a class of electromagnetic stimuli characterized by low frequency (1 to 80 Hz) and intensity ranging between 50 μT to 50 mT [26]. They were first FDA approved for the treatment of bone non-unions and delayed unions in the 1980s, and, since then, the growing interest in their mechanisms and abilities lead to a great number of scientific investigations. Indeed, the in vitro results confirmed their effectiveness in stimulating osteoblast and inhibiting osteoclasts activity [27, 28]. The mechanism underlying the effect of PEMFs on the biological tissue is still under debate, but many hypothesis and evidences were described. Patterson and colleagues reported the activation of phosphatidylinositol-3-kinase (PI3K) and mTOR (mammalian target of rapamycin) pathway, leading to the transcription of growth factors of the TGFβ family such as BMP-4 [29]. Intracellular calcium (Ca^2+^) concentration was also addressed as effector of PEMFs biological activity, in correlation with plasma membrane potential and currents [30–37]. Moreover, PEMFs are able to induce increase in the expression of adenosin A_2A_ receptors and integrinβ, influencing the related intracellular pathways with roles in anti-inflammatory and differentiation processes [38, 39] (Fig. 1).

The application of PEMFs to mesenchymal stem cells of different origins demonstrated their ability in the modulation of cell cycle and enhancement of differentiation. MSCs isolated from human bone marrow-derived (hBMSCs) were the most extensively adopted cells for this kind of experiments, and most of the studies reported an increased cell proliferation after PEMFs stimulation [40–43], as well as an increase of early stage markers of osteoblastic differentiation. In particular, many studies used PEMFs as adjuvant element, together with osteoinductive medium. In this experimental settings, increase in alkaline phosphatase (ALP) production, collagen type I and Runt-related transcription factor 2 (RUNX2) expression, and release of growth factors of the TGFβ family, such as BMP-2, were reported [41, 42, 44, 45]. On the other hand, their influence on the mineralization phase of osteogenic differentiation was controversial. Some studies reported an increased deposition of Ca^2+^ rich extracellular matrix [42, 44, 45], while others indicated that this late phase of osteogenic differentiation was not influenced by PEMFs [46]. Differences in each experimental setting could explain the discordant reports. In fact, in these studies different types of stimulation, in term of field intensity, frequency, and time of exposure were used. Moreover, other parameters such as the seeding density could produce different biological effects in the same cell type [47–50]. Despite these differences, taken together the reported data support the idea that PEMFs could enhance proliferation and osteodifferentiation of hBMSCs. Similarly, in combination with chondrogenic inductive medium, PEMFs stimulation was able to accelerate the hypertrophic cell differentiation, increasing deposition of collagen type I and X, and then osteochondral ossification in a 3D in vitro culture of rat BMSCs [51].

Other human cell types such as adipose derived stem cells (ASCs), tendon stem progenitor cells (TSPCs), amniotic epithelial cells (AECs), and umbilical cord MSCs (WJ-MSCs) were treated with PEMFs with similar results. hASCs proliferation and survival were enhanced by PEMFs treatment [52]. Moreover, in combination with chondrogenic inductive medium, PEMFs were able to increase ASCs chondrogenic differentiation, in terms of expression of Sox9, collagen type I and II, aggrecan and osteocalcin, as well as mineralized matrix, and glycosaminoglycans deposition [53]. Chondrogenic differentiation capacity and proliferation of WJ-MSCs were also enhanced by PEMFs exposure [54], while PEMF-treated AECs were more prone to differentiate toward osteogenic lineage with respect to unexposed cells [39].

TSPCs, a tendon cell subpopulation that possess all the features of MSCs [55], exposed to different PEMFs treatments, resulted in the increased expression of tenogenic markers, such as collagen type I, scleraxis, VEGF, IL-10 and TGFβ. Moreover, a slight increase in cell proliferation was observed in the same experimental setting [56, 57]. The anti-inflammatory effect of PEMFs was reported also in other cell types, such as rat BMSCs, where they were able to reduce the production of IL-1β and TNFα in a pathological model [27].

The results described in this section support the hypothesis that PEMFs could enhance the tissue homeostatic activity of MSCs. Indeed, cell proliferation and differentiation are the two main events occurring in the physiological tissue homeostasis process, in order to replace cells lost in a typical degenerative pathology. Moreover, the ability to enhance the release of growth factors, such as TGFβ and BMP-2, and to reduce anti-inflammatory agents, underlines the role of PEMFs in the instauration of a regenerative microenvironment within the tissues.

## Extracorporeal shock wave therapy

Since its original applications as urological lithotripsy, extracorporeal shock wave therapy (ESWT) has been applied in the musculoskeletal field as orthotripsy (mainly tendinopathies and bone regenerative disorders) and regenerative medicine as well [58, 59].

The mechanisms of action of ESWT, when applied in non-urological indications, are not related to the direct mechanical effect but to the different pathways of biological reactions that derive from those acoustic stimulations, through the so-called “mechanotransduction” mechanism. Therefore, the “mechanical model” of urological lithotripsy has been substituted by a “biological” one, also supported by the current knowledge in mechanobiology, an emerging multidisciplinary field of science that investigates how physical forces and changes in cell/tissue mechanics can influence the tissue development, physiology and diseases. Although some details are still under investigation, it is known that ESWT are able to relieve pain, reduce inflammation, and induce neoangiogenesis and stem cell activities, thus improving tissue regeneration and healing [58, 60].

Indeed, early researches on this topic showed that ESWT affected, according to the amount of energy and the number of pulses, the growth ratio of bone marrow osteoprogenitor cells, forming colony-forming unit-osteoprogenitors (CFU-O) and bone nodules related to the induction of TGFβ1 molecule [61].

Subsequent experimental results revealed that these effects included the regulation of submembrane redox reactions elicited by early O_2_ production for tyrosine kinase-mediated ERK activation, resulting in phosphorylation of CBFA1 (core-binding factor alpha1), the transcription factor for osteoblastic differentiation, and osteogenesis (Fig. 1). The evidence that shock waves triggered the growth and maturation of osteoprogenitor cells through the pathway of mechanotransduction, prefigured following models for ex vivo extension of the mesenchymal stem cells [62]. Moreover, in a more recent work, human BMSCs exposed to shock waves showed increased proliferation and migration capacity [63].

Nevertheless, the interest on the use of ESWT in regenerative medicine arose from the studies of their application in ischemic lesions, where, besides the angiogenic and anti-inflammatory effect, emerged the hypothesis of an action addressed to the proliferation, recruitment, migration, and homing of progenitors cells in the sites of lesion [64, 65]. In fact, reversible modification of the cytoskeleton was observed in ESW-treated cells, whose final effect, through nuclear transcription activation, is a series of specific biochemical pathways, related to local tissue turnover [66].

Indeed, already in 2005, it was demonstrated that pretreatment of hematopoietic stem cells with high energy shock waves significantly improved early progenitor cell expansion, after short-term suspension culture, prefiguring a new way to manipulate these cell populations [67].

In this perspective, MSCs, as a main target during regenerative pathways, could play a key role in orchestrating all the processes activated by shock waves [58].

Recently, basic science about shock waves was enriched of articles, describing their “regenerative effect” in many different tissues, other than the bone.

In order to elucidate the ESW-mediated clinical benefits, Leone et al., by studying TSPCs explanted from five healthy semitendinosus and five ruptured Achilles tendons found that the clonogenic potential was maintained only in cells derived from healthy donors. Moreover, ESW application significantly accelerated hTSPCs differentiation, thus suggesting that the clinical benefits of ESWT may be ascribed to increased efficiency of tendon repair after injury [68]. Indeed, these observations were confirmed in a different in vitro setting, where, TSPCs treated with ESW showed increased proliferation, expression of tendon specific markers (scleraxis and collagen type I) and production of growth factors (VEGF, TGFβ) and cytokines (IL-10), consistently with the instauration of a regenerative and anti-inflammatory microenvironment [69]. Very recently, Rinella and co-authors were able to demonstrate how ESWT would inhibit the development of a myofibroblast phenotype in human ASCs. Functionally, stem cells acquire a more fibroblast-like profile characterized by a low contractility and a high migratory ability, related to a reduced expression of integrin alpha 11, a major collagen receptor in fibroblastic cells, involved in myofibroblast differentiation. In other words, this in vitro study shows that ESW can control stem cell differentiation toward myofibroblasts and, consequently, sustain their use as a therapeutic approach in reducing the risk of skin and tissue fibrosis [70]. Other authors showed that ESWT enhances ASCs production of osteogenic markers, such as RUNX2, ALP, and mineralized matrix, but, at the same time, they could increase the production of reactive oxygen species (ROS) [71].

Very interestingly, Schuh et al., showed that human and rat ASCs respond strongly to repetitive shock wave treatments in vitro, resulting in maintenance and significant increase of mesenchymal markers (CD73, CD90, CD105), differentiation capacity toward the osteogenic and adipogenic lineage, as well as toward Schwann-cell like cells even after extended time in vitro. In their study, Schuh et al. concluded that ESWT might be a promising tool to improve ASCs quality for cell therapy in various tissue engineering and regenerative medicine applications [72].

From a general point of view, the preconditioning of MSCs by ESW appears to improve their therapeutic performance without issues about the extensive manipulation that characterizes others approaches, such as genetic manipulations [63].

## Conclusions

Recently, the development of stem cell-based therapies has been quickly spreading in numerous disease areas, including musculoskeletal disorders. Whether taken from the bone marrow or adipose tissue or umbilical cord, even though they can be isolated from almost all body compartments, the mesenchymal stem cells seem to show a remarkable potential for their direct use in musculoskeletal tissues repair.

In vivo observations laid the ground for the hypothesis that mechanical factors could play a major role in regulating the development and repair of the musculoskeletal tissues. Undoubtedly, these studies have greatly improved our knowledge about MSCs response to specific stimuli and the isolation of any single variables has facilitated the discovery of several mechanotransductive mechanisms. However, the in vivo environment is definitely much more complex. Cells are exposed to several intrinsic and extrinsic mechanical cues simultaneously and to the interaction between the biophysical and biochemical environment. Beside their well-known effectiveness on the musculoskeletal tissues, the idea of a direct stimulation of the resident MSCs by shock waves and pulsed electromagnetic fields has been a recent and intriguing hypothesis. Preliminary experimental data seem to confirm that these stimuli are able to enhance MSCs homeostatic activity and, thus, their role in preventing and counteracting musculoskeletal degenerative processes.

Tissue engineering could be another key application of mechanically treated MSCs, even though there are still many technical challenges associated with isolating, expanding, differentiating, and preconditioning MSCs for subsequent implantation into the degenerate joints and tissues. Moreover, the MSCs could be exposed to abnormal physical loads in anatomical structures that have already been biomechanically compromised. Another aspect that requires a deeper knowledge is ascertaining if the in vitro cultivated cells need predifferentiation or a sort of preconditioning prior to implantation into the damaged tissues, with the aim to enhance cell survival and matrix formation. Mechanical stimulation could be a low-cost, useful tool in this respect. Nevertheless, adequate requisite and design of MSCs cell-supporting biomaterials that will resist the enzyme-rich, mechanically load microenvironments of the degenerated musculoskeletal tissues also remain to be better clarified.

In summary, MSC-based therapies could offer a remarkable potential to change dramatically the treatment of cartilage defects, tendon degeneration, and several other musculoskeletal disorders. The advances discussed in this review highlight the progress being made toward clinical translation of such therapeutic approaches and which might be further enhanced by the aid of mechanotransduction. However, a number of technical problems and conceptual challenges still need to be addressed as research proceeds.

## Abbreviations

AECs: Amniotic epithelial cells
ALP: Alkaline phosphatase
ASCs: Adipose-derived stem cells
BMSCs: Bone marrow-derived mesenchymal stem/stromal cells
BMP-2: Bone morphogenetic protein 2
BMP-4: Bone morphogenetic protein 4
CBFA1: Core-binding factor alpha1
CD: Cluster of differentiation
CFU-O: Colony-forming unit-osteoprogenitors
DAMP: Damage associated molecular pattern
ESWT: Extracorporeal shock wave treatment
IL-10: Interleukine 10
IL-1β: Interleukine 1 beta
MSCs: Mesenchymal stem/stromal cells
mTOR: Mammalian target of rapamycin
PAMP: Pathogen associated molecular pattern
PEMFs: Pulsed electromagnetic fields
PSCs: Perivasular stem cells
ROS: Reactive oxygen species
RUNX2: Runt-related transcription factor 2
Sox9: SRY-Box 9
TGFβ: Transforming growth factor beta
TSPCs: Tendon stem progenitor cells
VEGF: Vascular endothelial growth factor
WJ-MSCs: Wharton’s jelly derived mesenchymal stem/stromal cells
